# Examining Whether Patient Portal and Video Visit Use Differs by Race and Ethnicity Among Older Adults in a US Integrated Health Care Delivery System: Cross-Sectional Electronic Health Record and Survey-Based Study

**DOI:** 10.2196/63814

**Published:** 2024-11-07

**Authors:** Nancy P Gordon, Chelsea Yin, Joan C Lo

**Affiliations:** 1 Kaiser Permanente Division of Research Kaiser Permanente Northern California Pleasanton, CA United States; 2 Department of Adult and Family Medicine Kaiser Permanente Oakland Medical Center Oakland, CA United States; 3 Kaiser Permanente Division of Research and The Permanente Medical Group Kaiser Permanente Northern California Pleasanton, CA United States

**Keywords:** patient portal use, video visit use, older adults, racial and ethnic differences, telehealth, mobile phone

## Abstract

**Background:**

Health care systems are increasingly encouraging patients to use patient portals and participate in video visits. However, there is limited information about how portal use differs among older adults.

**Objective:**

This study aimed to understand how patient portal and video visit use differed by age, race, and ethnicity among older adult patients with access to the same digital health resources.

**Methods:**

This cross-sectional study used electronic health record and survey data for adults aged 65 to 85 years who were members of a large Northern California health care delivery system throughout 2019 and 2020. The electronic health record cohort (N=471,152) included 320,686 White, 35,892 Black, 44,922 Latino, 20,786 Chinese, 28,732 Filipino, 8473 South Asian, 6716 Japanese, 2930 Vietnamese, and 2015 Korean adults. Racial and ethnic group and age group (65 to 75 years vs 76 to 85 years) differences in having a patient portal account by December 2020, the performance of 2 portal activities (sending ≥1 message to a clinician in 2019 or 2020 and viewing ≥1 laboratory test result in 2020), and having ≥1 video visit during 2020 were examined. Modified log-Poisson regression was used to examine prevalence ratios for portal and video visit use, comparing racial and ethnic groups to White adults and Asian ethnic groups to Chinese adults after adjusting for sex and age. Data from a 2020 member survey were used to compare internet use factors among 2867 White, 306 Black, 343 Latino, 225 Chinese, and 242 Filipino adults.

**Results:**

Black, Latino, and Filipino adults were less likely to have a patient portal account than White adults, and Filipino adults were less likely to have a patient portal account than Chinese adults. Black, Latino, Filipino, Korean, Vietnamese, and South Asian adults were less likely to have sent messages and viewed test results than White adults, while Chinese and Japanese adults’ use of these features was similar to that of White adults. Filipino, Vietnamese, and Korean adults were less likely to have performed the aforementioned activities than Chinese adults. Video visit use was lower among Black and Latino adults and higher among Chinese and South Asian adults compared with White adults (aged 76 to 85 years) and lower among Filipino, Korean, and Vietnamese adults compared to Chinese adults. Survey data suggested that underlying differences in internet use may partially explain the lower use of messaging by Black, Latino, and Filipino adults compared with White and Chinese adults.

**Conclusions:**

Patient portal and video visit use differed by race, ethnicity, and age group among older adult patients with access to the same patient portal. Internet use factors may contribute to these differences. Differences in patient portal and video visit use across Asian subgroups underscore the importance of disaggregating use data by Asian ethnicity.

## Introduction

### Background

Clinicians and patients are increasingly communicating and accessing health information electronically using patient portals [1,2]. Patient portals enable patients and their health care proxies to access selected health information in the patient’s electronic health record (EHR), exchange messages with health care providers, view laboratory test results, order prescription refills, and schedule appointments, among other health care–related activities [3]**.** These patient portal activities, grouped under the umbrella of asynchronous telehealth, can be conveniently undertaken by both patients and clinicians at any time and from any location with internet service. Video visits, a form of synchronous telehealth, enable patients and health care providers to interact face to face from different locations. Video visits improve access and convenience for patients and can reduce clinic visit–related costs (eg, transportation and copays) [4-8]. Video visits also help address outpatient health care worker shortages and reduce physician burnout [9,10], although evidence is mixed for these outcomes and whether they ultimately reduce health care delivery costs [11]. Video visit use greatly increased in 2020 during the COVID-19 public health emergency as delivery of health care shifted from in-person clinic visits to video and phone visits when in-person care was not necessary [12-15]. During this time, Medicare reimbursed clinicians for virtual visits at the same rate as in-clinic visits, and many health insurers temporarily waived copays for video visits for commercially insured members [16,17].

Patient portals have the potential to enhance the patient-clinician relationship; improve health status; and increase self-management behaviors, including adherence to therapy [18]. Older adults stand to benefit a great deal from the ability to use patient portals and video visits. As a demographic group, they have a high burden of chronic health problems [19], with concomitant higher use of prescription medications, laboratory tests, medical visits, and the need to communicate with their health care providers outside of scheduled visits. Portals provide a convenient way to send and receive detailed communications rather than leaving brief phone messages with support staff during regular business hours. Portals also give patients and their proxies 24/7 access to viewing laboratory test results, appointment scheduling, ordering prescription refills, and viewing medical and visit history. Video visits can make it easier for older adults to access health care when in-clinic examinations or tests are not needed, especially for those who are frail, have mobility limitations, or have transportation-related difficulties. Video visits can also enable spouses and other caregivers to participate in scheduled visits in a way that is not always possible in clinic-based visits.

While the use of patient portals and video visits has been increasing among older adults, it remains substantially lower among middle-aged and younger adults. Philpot et al [20] used the term "digital determinants of health" to describe barriers to the use of patient portals and video visits, including lack of access to an internet-enabled device and webcam (for video visits); lack of high speed or broadband internet at home; lack of digital health literacy, skill, or confidence in the ability to use digital health technologies; concerns about privacy and security; cognitive or physical impairment that limits the use of a computer or touchscreen devices, internet, or complex websites; and lack of technical assistance from others [3,21-24]. In several studies, successful use of digital health tools by older adults depended on older adults’ motivation and the support that they received [25].

While several studies have documented racial and ethnic group differences in patient portal and video visit use among general and disease-specific populations of US adults [26-31], there is a lack of contemporary data regarding racial and ethnic group differences in use of patient portal functions and video visits among US adults aged ≥65 years in health care systems that provide access to patient portals and video visits for all patients. Using 2013 to 2014 EHR and survey data to examine patient portal use and factors associated with use among Kaiser Permanente Northern California (KPNC) health plan members aged 65 to 79 years, our team previously observed that Black, Latino, and Filipino adults were less likely than White and Chinese adults to have to a patient portal account; to have signed into the patient portal at least once; and to have used the patient portal to send a secure message, view laboratory test results, or order prescription refills at least once by the end of 2013 [31]. In the same study, a mailed survey conducted in a random subset of health plan members in winter 2013-2014 found that Black, Latino, and Filipino, but not Chinese, adults were less likely than White adults to have access to digital tools, to have experience performing a variety of web-based tasks, to feel they were capable of going on the internet to obtain health information, and to be interested in using web-based health information modalities [31]. However, this early study was limited to those from 5 racial and ethnic groups aged 65 to 79 years and did not examine racial and ethnic differences in patient portal use among younger or older subsets. In addition, video visit use was not examined because video visits were not widely offered until 2020.

Since the start of the COVID-19 pandemic, health care providers have increasingly transitioned to the use of patient portals as a primary platform for sharing laboratory test results and other health care–related information with patients, for bidirectional messaging with patients to address health concerns rather than having phone calls, and for patients to self-schedule nonurgent appointments and order prescription medication refills on the web rather than through a call center. This transition could potentially exacerbate existing disparities in health information access and disparities in access to and use of digital health tools [32], the latter recently termed as gaps in digital health equity [33] or health “techquity” [34]. Given well-documented evidence of racial and ethnic disparities in access to and use of the internet [27,35-38], it is important to understand the extent to which racial and ethnic differences in the use of patient portal functions and video visits occur among older adults. Furthermore, as ethnic diversity increases within the older adult population, especially within the Asian population, it is important to learn how the use of patient portals and video visits may vary across Asian ethnic groups, a currently understudied segment of the US population.

### This Study

Our study addresses the aforementioned knowledge gaps by analyzing contemporaneous EHR and survey data for older members of a large Northern California health care delivery system. We first documented racial and ethnic differences in health plan patient portal and video visit use among adults aged 65 to 85 years whose preferred language for written materials in the EHR was English, including comparisons among Asian ethnic groups. We then used data from a health plan member survey for a subset of adults in the EHR cohort to examine educational attainment and internet use factors that might contribute to the differences in patient portal use across racial and ethnic subgroups.

## Methods

### Ethical Considerations

This work was conducted in accordance with procedures approved by the Kaiser Permanente Northern California (KPNC) Institutional Review Board. For the EHR cohort study (IRBnet #1279536-6), the KPNC Institutional Review Board waived the requirements to obtain informed consent and privacy rule authorization for use and disclosure of protected health information. The secondary analysis of the 2020 Member Health Survey data and linkage of survey respondent data to EHR data for the survey cohort study fell within the scope of the original 2020 Member Health Survey research protocol approved by the KPNC Institutional Review Board (IRBnet #1539606-3) which waived the requirement to document informed consent and privacy rule authorization for use and disclosure of protected health information.

### Setting

KPNC is an integrated health care delivery system that provides primary and specialty health care, laboratory testing, and pharmacy services to a racially, ethnically, and sociodemographically diverse membership that includes >3.2 million adults who mostly reside in the Greater San Francisco Bay Area, Sacramento area, Silicon Valley, and the Central Valley. The KPNC adult membership has been shown to be very similar to the insured population of Northern California adults with regard to sociodemographic and health characteristics [39,40]. Since 2007, KPNC has maintained a patient portal as part of its website that enables members to communicate with their health care team and member services using secure messages; view laboratory test results, immunizations, past office visits, prescriptions, and health conditions; order prescription refills; and schedule or cancel nonurgent appointments [41]. To use the patient portal, members must register for and activate a patient portal account and sign onto the portal with their user ID and password to perform portal activities. During the study period (2019-2020), members were able to set up and activate their patient portal account or reset their password through the health plan’s website or a Kaiser Permanente app for immediate use. Some patient portal content was available in Spanish but not in other non-English languages.

### Study Population

#### EHR Cohort

The EHR study cohort was a subset of a 2019 EHR-based research cohort created to study demographic differences in health status and health care use in a very large (>2.4 million) cohort of adults aged 25 to 89 years who were KPNC members throughout 2019. More detail on how the demographic cohort was created can be found in an earlier publication [42]. The cohort for this study (N=471,152) comprised 320,686 (68.06%) non-Hispanic White (White); 35,892 (7.62%) African American or Black (Black); 44,922 (9.53%) Hispanic or Latino (Latino); 28,732 (6.1%) Filipino; 20,786 (4.41%) Chinese; 8473 (1.8%) South Asian; 6716 (1.43%) Japanese; 2930 (0.62%) Vietnamese; and 2015 (0.43%) Korean men and women who were aged 65 to 85 years on December 1, 2020. All adults had English as their preferred written language in the EHR and were KPNC members for all 24 months from 2019 to 2020.

#### Survey Cohort

The KPNC Member Health Survey is an English-only self-administered (mail or on the web) research survey that collects information about sociodemographic and health characteristics and the use of digital information technology. Survey details are published elsewhere [43,44]. Because the 2020 Member Health Survey sample was drawn from the same source cohort used to create the EHR cohort for this study, the survey cohort is a subset of the larger EHR cohort. The survey sample included 2867 White, 306 Black, 343 Latino, 225 Chinese, and 242 Filipino adults aged 65 to 85 years who answered survey questions pertaining to internet use factors. Only the 2 largest Asian ethnic groups are represented as the other 4 Asian ethnic groups had too few respondents to examine as a subgroup. Of the 52.9% (4269/8065) of adults aged 65 to 85 years in these 5 racial and ethnic groups who responded to the survey, a total of 93.9% (3983/4269) answered internet use questions.

### Study Variables

#### EHR Cohort

A description of the methods used to assign race and ethnicity, including the use of language preference in the EHR, member survey sources, and surname assignment can be found in an earlier 2019 publication appendix [42]. Age on December 1, 2020 (integer variable), was determined from the date of birth. Sex was restricted to male or female as indicated in the EHR. Chronic cardiovascular conditions (diabetes, hypertension, ischemic heart disease, and heart failure) were assigned using International Classification of Disease Tenth Revision outpatient visit and problem list diagnoses from 2018 to 2019.

We used data abstracted from KPNC EHRs to create the following 4 variables: (1) indication of an activated patient portal account by December 1, 2020; (2) history of having sent ≥1 secure message to a clinician during 2019 or 2020; (3) history of viewing laboratory test results during 2020 if ≥1 laboratory test result was released during 2020; and (4) history of having ≥1 video visit during 2020 if the person had ≥1 ambulatory visit in a department that offered video visits during 2020. Because sending a secure message was not restricted to a subset of the population that experienced a particular event (ie, having a laboratory test released during the year), we ascertained this outcome over a larger 2-year window to allow more time for patients to have sent a secure message. Video visit use was only examined for 2020 due to its infrequent use before the onset of the COVID-19 pandemic emergency in March 2020. Because an activated patient portal account was necessary for sending secure messages and viewing laboratory test results, we examined differences in the use of these patient portal functions by all patients and by patients who had an activated portal account by the end of 2020. An activated patient portal account was not required for a video visit. Starting in March 2020, most outpatient visits were conducted by video or phone due to the COVID-19 pandemic.

#### Survey Cohort

We examined 6 variables from survey data and 3 variables from EHR data. Educational attainment was examined as a 3-level variable: high school graduate or less, some college or associate degree, or bachelor’s degree or higher. Use of the internet was based on the question, “Do you use the internet (go online) to get information, watch videos, fill out forms, pay for things, etc.?” Response options included “Yes, I use it by myself;” “Yes, but someone else helps or uses it for me;” or “No, I don’t use the internet.” Access to a desktop, laptop, or tablet computer was based on a “yes” to the question, “Do you have access to a desktop, laptop, or tablet computer that you can (or could) use to go online (use the internet)?” and access to an internet-capable device was assigned based on having a computer, tablet, or smartphone. Internet users were asked, “Can you easily print information/forms you get from the internet?” Respondent data were linked to EHR patient portal use data: having an activated patient portal account by June 2020, sending ≥1 message using the patient portal between January 2019 and June 2020, and having a video visit in 2020.

### Statistical Analyses

#### EHR Cohort Analyses

All analyses were performed using SAS (version 9.4; SAS Institute). For each patient portal use variable and video visit use, we examined portal use statistics for the 9 racial and ethnic groups, overall, and for those aged 65 to 75 and 76 to 85 years within the racial and ethnic groups. Chi-square tests were used to compare demographic groups, with the largest group (White adults) serving as the reference. Chinese adults were used as the reference group for comparisons among the 6 disaggregated Asian ethnic groups. Previous KPNC research on the use of digital information technologies and patient portal functions found that Chinese adults were more likely to use the internet, obtain health information from websites, and use the patient portal than Filipino adults [31,35].

Because the racial and ethnic groups being compared were very large, very small differences in percentages that were statistically significant were not always meaningful. We thus made an a priori decision to use a between-group absolute difference of ≥5 percentage points as the threshold for meaningful difference. Hence, for descriptive analyses pertaining to the EHR cohort, all demographic differences described in the Results section met the criterion of an absolute difference of ≥5 percentage points and a chi-square *P* value of <.05 for differences in proportions. Modified log-Poisson regression models were used to estimate adjusted prevalence ratios (aPRs) with 95% CIs for each portal use outcome using White or Chinese groups as reference and controlling for sex and age as a continuous variable. As the portal use outcomes being modeled were not rare events and we were comparing group-level prevalence rather than individual likelihood of portal outcome use, the aPR is a more appropriate measure of association than an adjusted odds ratio derived from a logistic regression model, which can inflate the strength of association [45].

#### Survey Cohort Analyses

Survey respondents were assigned poststratification population weighting factors so that the analytic weighted sample would more closely approximate the age, sex, and racial composition of adults aged 65 to 85 years with English written language preference in the 2019 KPNC membership (ie, the same population that was used for the EHR cohort). To prevent variance inflation, population weights were normalized (rescaled) so that the number in the analytically weighted sample would be equivalent to the number of survey respondents while maintaining the same age-sex-racial-ethnic composition of the population-weighted sample. Normalized weighting factors were created by multiplying the population weighting factors by the reciprocal of the sum of the survey population weights divided by the total number of survey respondents.

All analyses were performed using weighted survey data with SAS (version 9.4). Cross-tabular analyses were used to produce estimates of internet use, device access, and portal use for each racial and ethnic group. To test for racial and ethnic group differences, we used modified log-Poisson regression models that adjusted for age (5-year age group) and sex (female vs male) and for age, sex, and education (≤ high school graduate and some college or associate degree vs ≥ bachelor’s degree). Modified log-Poisson regression models were also used to examine the association of internet use status with patient portal use after controlling for demographic characteristics, reporting aPRs with 95% CIs.

## Results

### EHR Cohort

#### Demographic and Health Characteristics of the EHR Study Cohort

As presented in Table 1, all 9 racial and ethnic groups had approximately the same mean age, but Chinese and South Asian groups had higher proportions of adults aged 65 to 75 years compared to White adults. The South Asian group had a higher proportion of men. Black, Latino, Filipino, and South Asian groups had higher proportions of adults with a chronic cardiovascular condition (diabetes, hypertension, ischemic heart disease, or heart failure). Among the Asian ethnic groups, compared to the Chinese group, the Japanese and Korean groups had higher proportions of adults aged 65 to 75 years, whereas the Filipino and South Asian groups had higher proportions of adults with chronic cardiovascular conditions.

**Table 1:** Characteristics of racial and ethnic groups in the electronic health record study cohort of adults aged 65 to 85 years (N=471,152).

Characteristic			White (n=320,686)		Black (n=35,892)		Latino (n=44,922)		Chinese (n=20,786)		Filipino (n=28,732)		Japanese (n=6716)		Korean (n=2015)		Vietnamese (n=2930)		South Asian (n=8473)
**Sex (%)**																			
	Male	45.1		40.7		44.2		47.7		41		41.4		40.9		51.4^a^		57.2^a^	
	Female	44.9		59.3		55.8		52.3		59.0		58.16		59.1		48.6		42.8	
**Age, Mean (SD)**			73.5 (5.2)		73.1 (5.3)		73.1 (5.3)		72.8 (5.2)		73.0 (5.2)		73.8 (5.4)		73.7 (5.1)		72.1 (4.8)		72.5 (5.0)
	65 to 75 (%)	66.3		68.7		68.4		72.8^a^		70		63.4^b^		65.4^b^		76		73.2^a^	
	76 to-85 (%)	33.7		31.3		31.6		27.2		30		36.6		34.6		24		26.8	
≥1 chronic cardiovascular condition^c^ (%)			63.8		83.7^a^		73.1^a^		63.4		84.9^a,b^		67.4		64.4		66.5		77.9^a,b^

^a^Racial-ethnic group differed from White adults by ≥5 percentage points and the difference was statistically significant at *P*<.001.

^b^Asian ethnic group differed from Chinese adults by ≥5 percentage points and the difference was statistically significant at *P*<.001.

^c^Chronic cardiovascular conditions included diabetes, hypertension, ischemic heart disease, or heart failure diagnosis.

#### Patient Portal Account Status by the End of the Year 2020

As presented in Table 2, ≥80% (range 81%-94%) of adults aged 65 to 85 years in each of the 9 racial and ethnic groups had an activated patient portal account by the end of 2020. The lowest percentage was seen for Black adults aged 76 to 85 years (73%), and the highest percentage (95%) was seen for White, Chinese, and Japanese adults aged 65 to 75 years. In all age groups, Black, Latino, and Filipino adults were less likely to have an activated portal account than White adults, and Filipino adults were less likely to have an activated portal account than Chinese adults. After adjusting for age and sex, Black, Latino, Filipino, and Vietnamese adults, respectively, were 14%, 10%, 7%, and 5% less likely than White adults to have a patient portal account, and Filipino adults were 7% less likely than Chinese adults to have a patient portal account (Figure S1 in Multimedia Appendix 1). More than 90% of adults (range 92%-99%) with an activated portal account by December 2020 also had an activated account in 2019 (data not shown).

**Table 2:** Racial and ethnic group differences in patient portal and video visit use from 2019 to 2020 by adults aged 65 to 85 years, overall and by age group.

		White	Black	Latino	Chinese	Filipino	Japanese	Korean	Vietnamese	South Asian
**Had a patient portal account by the end of 2020^a^**										
	All aged 65 to 85 years, n (%)	320,686 (94.1)	35,892 (80.8)^b,c^	44,922 (85.4)^b,c^	20,786 (93.9)	28,732 (87.3)^b,c,d^	6716 (93.1)^c^	2015 (89.4)	2930 (91.7)	8473 (93.6)
	Those aged 65 to 75 years, n (%)	212,545 (95.6)	24,647 (84.5)^b^	30,705 (87.7)^b^	15,126 (95.1)	20,120 (89.1)^b,d^	4257 (95.4)	1318 (90.1)^b,d^	2227 (92.4)	6204 (93.8)
	Those aged 76 to 85 years, n (%)	108,141 (91.2)	11,245 (72.6)^b^	14,217 (80.3)^b^	5660 (90.6)	8612 (83.2)^b,d^	2459 (89.3)	697 (88.1)	703 (89.5)	2269 (93.1)
**Sent ≥1 secure message in 2019 or 2020^a^**										
	All aged 65 to 85 years, n (%)	320,686 (80.2)^c^	35,892 (54.2)^b,d^	44,922 (60.8)^b,d^	20,786 (76.3) ^c^	28,732 (59.9)^b,c,d^	6716 (75.8)^c^	2015 (61.1)^b,d^	2930 (66.7)^b,c,d^	8473 (74.5)^b^
	Those aged 65 to 75 years, n (%)	212,545 (82.9)	24,647 (58.7)^b^	30,705 (64.2)^b^	15,126 (78)	20,120 (62.9)^b,d^	4257 (79.5)	1318 (62.3)^b,d^	2227 (68.3)^b,d^	6204 (74.5)^b^
	Those aged 76 to 85 years, n (%)	108,141 (74.8)	11,245 (44.4)^b^	14,217 (53.5)^b^	5660 (71.8)	8612 (53.1)^b,d^	2459 (69.3)^b^	697 (59)^b,d^	703 (61.6)^b,d^	2269 (74.7)
**Viewed laboratory results ≥1 time in 2020 (adults with ≥1 laboratory result released in 2020)^a^**										
	All aged 65 to 85 years, n (%)	277,726 (84.4)^c^	31,145 (59.2)^b,d^	39,582 (66.8)^b,d^	17,743 (83.7)^c^	25,087 (68.9)^b,c,d^	5738 (83)^c^	1742 (71.4)^b,c,d^	2536 (76.4)^b,d^	7517 (80.9)
	Those aged 65 to 75 years, n (%)	186,871 (87.1)	21,781 (63.8)^b^	27,471 (70.6)^b^	13,195 (85.3)	18,057 (71.8)^b,d^	3744 (86.4)	1163 (73.5)^b,d^	1958 (77.9)^b,d^	5619 (81.1)^b^
	Those aged 76 to 85 years, n (%)	90,855 (78.8)	9364 (48.6)^b^	12,111 (58.2)^b^	4548 (78.9)	7030 (61.2)^b,d^	1994 (76.4)	579 (67)^b,d^	578 (71.5)^b,d^	1898 (80.2)
**Had ≥1 video visit in 2020 (adults with ≥1 ambulatory visit in 2020)^a^**										
	All aged 65 to 85 years, n (%)	286,687 (48)^b^	32,374 (43.6)^b^	40,815 (43.4)^b^	17,902 (52.8)^b^	25,678 (47.1)^c^	5778 (49.6)^b^	1791 (44.4)^b,c^	2569 (46.9)^c^	7688 (54.4)^a^
	Those age 65 to 75 years, n (%)	187,475 (50.6)	22,132 (46.2)	27,593 (46.1)	12,848 (54.4)	17,905 (47.9)^c^	3562 (53)	1150 (46.3)^c^	1930 (47.5)^c^	5615 (54.3)
	Those aged 76 to 85 years, n (%)	99,212 (42.9)	10,242 (37.9)^a^	13,222 (37.8)^a^	5054 (48.8)^a^	7773 (45.3)^c^	2216 (44)	641 (41)^c^	639 (45.2)	2073 (54.7)^a,c^

^a^n: total number of adults in this age subgroup used as the denominator to calculate the percentage.

^b^Meaningful difference between this racial-ethnic group and White adults based on ≥5 percentage point difference that was statistically significant at *P*<.05. Differences remained statistically significant after controlling for age and sex.

^c^Meaningful difference between those aged 65 to 75 years and 76 to 85 years within this racial or ethnic group based on ≥5 percentage point difference that was statistically significant at *P*<.05. Differences remained statistically significant after controlling for age and sex.

^d^Meaningful difference between this Asian ethnic group and Chinese adults based on ≥5 percentage point difference that was statistically significant at *P*<.05. Differences remained statistically significant at *P*<.05 after controlling for age and sex.

#### Use of Secure Messaging Through the Patient Portal During 2019 and 2020

There was substantial racial and ethnic variation in the use of secure messaging, ranging from a low value of 54% for Black adults to a high value of 80% for White adults. The lowest percentage was seen for Black adults aged 76 to 85 years (44%) and the highest percentage was seen for White adults aged 65 to 75 years (83%). Significant racial and ethnic differences in the percentage of adults who sent ≥1 secure message in 2019 or 2020 were seen in the overall 65- to 85-year age group and both age subgroups (Table 2). Black, Latino, Filipino, Korean, and Vietnamese adults in all 3 age groups and South Asian adults aged 65 to 85 years and 65 to 75 years were less likely than White adults to have sent a secure message during that time, and in all but the South Asian group, adults aged 76 to 85 years were less likely to have sent a secure message than adults in the 65 to 75 years age group. Among Asian ethnic groups, Filipino, Korean, and Vietnamese adults in all 3 age groups were less likely than Chinese adults to have used secure messaging. After adjusting for age and sex, Black, Latino, Chinese, Filipino, Korean, Vietnamese, and South Asian adults, respectively, were 33%, 25%, 6%, 26%, 24%, 18%, and 8% less likely than White adults to have sent a secure message (Figure S2 in Multimedia Appendix 1). Filipino, Korean, and Vietnamese adults, respectively, were 21%, 19%, and 13% less likely than Chinese adults to have sent ≥1 secure message. The same racial and ethnic differences were seen in the subset of adults who had an activated portal account by the end of 2020, but some of the meaningfully significant age-specific differences within racial and ethnic groups in the full population were nonsignificant among those with activated accounts (Table 3).

**Table 3:** Racial and ethnic differences in patient portal use during 2019 and 2020 among adults aged 65 to 85 years, overall and by age group, restricted to adults who had an activated patient portal account by the end of 2020.

		White	Black	Latino	Chinese	Filipino	Japanese	Korean	Vietnamese	South Asian	
**Sent ≥1 secure message in 2019 or 2020^a^**											
	Overall cohort, aged 65 to 85 years, n (%)	301,855 (85.2)	28,991 (67.1)^b,c^	38,346 (71.2)^b,c^	19,511 (81.2)	25,090 (68.6)^b,c,d^	6255 (81.3)^c^	1801 (68.4)^b,d^	2687 (72.7)^b,d^	7931 (79.6)^b^	
	Aged 65 to 75 years, n (%)	203,219 (86.7)	20,832 (69.5)^b^	26,928 (73.2)^b^	14,385 (82)	17,922 (70.6)^b,d^	4060 (83.4)	1187 (69.1)^b,d^	2058 (74)^b,d^	5818 (79.4)^b^	
	Aged 76 to 85 years, n (%)	98,636 (81.9)	8159 (61.1)^b^	11,418 (66.4)^b^	5126 (79.1)	7168 (63.6)^b,d^	2195 (77.4)	614 (66.9)^b,d^	629 (68.7)^b,d^	2113 (80.2)	
**Viewed laboratory results ≥1 time in 2020 (adults with ≥1 laboratory result released in 2020)^a^**											
	Overall cohort, aged 65 to 85 years, n (%)	263,937 (88.8)	25,652 (71.9)^b,c^	34,263 (77)^b,c^	16,818 (88.2)	22,242 (77.6)^b,c,d^	5425 (87.7)^c^	1577 (78.7)^b,d^	2355 (82.3)^b,d^	7072 (85.9)	
	Aged 65 to 75 years, n (%)	180,024 (90.4)	18,691 (74.3)^b^	24,410 (79.4)^b^	12,655 (88.9)	16,260 (79.6)^b,d^	3610 (89.6)	1066 (80.1)^b,d^	1832 (83.2)^b,d^	5292 (86.1)	
	Aged 76 to 85 years, n (%)	83,913 (85.3)	6,961 (65.3)^b^	6961 (65.3)^b^	4163 (86)	5952 (72.1)^b,d^	1815 (83.7)	511 (75.7)^b,d^	9853 (71.3)^b^	1780 (85.5)	

^a^n: total number of adults in this age subgroup used as the denominator to calculate the percentage.

^b^Meaningful difference between this racial-ethnic group and White adults based on ≥5 percentage point difference that was statistically significant. Differences remained statistically significant at *P*<.05 after controlling for age and sex.

^c^Meaningful difference between those aged 65 to 75 years and 76 to 85 years within this racial or ethnic group based on ≥5 percentage point difference that was statistically significant at *P*<.05. Differences remained statistically significant after controlling for age and sex.

^d^Meaningful difference between this Asian ethnic group and Chinese adults based on ≥5 percentage point difference that was statistically significant at *P*<.05. Differences remained statistically significant after controlling for age and sex.

#### Viewed Laboratory Test Results Using the Portal During 2020

Among adults aged 65 to 85 years with ≥1 laboratory test result released in 2020, the percentage of those who viewed laboratory results using the portal ranged from 67% for Latino adults to 84% for White adults. The lowest percentage was seen for Black adults aged 76 to 85 years (49%) and the highest was seen for White adults aged 65 to 75 (87%). Black, Latino, Filipino, Korean, and Vietnamese adults in all age groups and South Asian adults aged 65 to 75 years were less likely to have viewed laboratory test results using the portal than White adults (Table 2). In all age groups, Filipino, Korean, and Vietnamese adults were less likely to have viewed laboratory results on the web than Chinese adults. In all racial and ethnic groups except Vietnamese and South Asian groups, adults aged 76 to 85 years were less likely to have viewed laboratory results using the portal than adults aged 65 to 75 years. Adjusting for age and sex, Black, Latino, Filipino, Korean, Vietnamese, and South Asian adults aged 65 to 85 years were 30%, 21%, 19%, 15%, 11%, and 5%, respectively, less likely than White adults to have viewed a laboratory test result using the portal during that year, and Filipino, Korean, and Vietnamese adults were 17%, 14%, and 9% less likely than Chinese adults to have done so (Figure S3 in Multimedia Appendix 1). The same racial and ethnic differences were seen among adults who had a portal account in 2020, except for the South Asian group (Table 3).

#### Video Visit During 2020

Among adults who had ≥1 ambulatory visit with a department that offered video visits during 2020, the percentages of those who used this modality ranged from a low value of 43% for Black and Latino adults to a high value of 54% for South Asian adults in the 65 to 85 years age group (Table 2). The lowest use was seen for Black and Latino adults aged 76 to 85 years (38%) and the highest (54%) for South Asian adults in all age groups and Chinese adults aged 65 to 75 years. Overall, South Asian adults were more likely than White adults to have had a video visit. Otherwise, differences between White adults and the other racial and ethnic groups were observed only in the 76- to 85-year age group (Black and Latino adults were less likely and Chinese and South Asian adults were more likely to have had a video visit during 2020). Filipino and Korean adults in all age groups and Vietnamese adults aged 65 to 85 years and 65 to 75 years were less likely to have had a video visit than Chinese adults, and South Asian adults aged 76 to 85 years were more likely to have had a video visit than Chinese adults. Adjusting for age and sex, Black, Latino, and Korean adults, respectively, were 10%, 10%, and 7% less likely, and Chinese and South Asian adults were 9% and 12% more likely than White adults to have had a video visit in 2020. After adjusting for age and sex, Filipino, Japanese, Korean, and Vietnamese adults, respectively, were 11%, 5%, 15%, and 12% less likely to have had a video visit than Chinese adults (Figure S4 in Multimedia Appendix 1).

### Survey Cohort

#### Racial and Ethnic Group Differences in Educational Attainment

Black and Latino adults were less likely than White and Chinese adults to have a bachelor’s degree or higher and more likely to have low educational attainment (≤ high school graduate), while educational attainment for Filipino adults was not significantly different from White or Chinese adults (Table 4). Chinese adults were more likely to have a higher educational attainment (at least a bachelor’s degree) than White adults. Among White, Black, and Filipino adults, older adults aged 76 to 85 years (vs those aged 65 to 75 years) were more likely to have low educational attainment.

**Table 4:** Comparison of educational attainment, digital information technology use, and patient portal use across White, Black, Latino, Chinese, and Filipino health plan members aged 65 to 85 with English language preference^a^.

					White (%)			Black (%)			Latino (%)			Chinese (%)			Filipino (%)
**Educational attainment**																	
	High school diploma or less			17.2^b,c^			22.7^b,c,d^			40.4^b,d^			10.7^d^			16.8^c^	
	Some college or associate degree			33.9			44.2			35.8			26.7			26.4	
	Bachelor’s degree or higher			48.9^b^			33.1^b,d^			23.8^b,d^			62.6^d^			56.7^e^	
**Uses the internet (alone or with help) to get information or watch videos (all adults)**					93^e^			83.7^b,d,e^			84.9^b,d,e^			94.3^e^			87.9^b,d,e^
	**Uses the internet without help**																
		All adults	86.2^e^			71.3^b,d,e^			71.9 ^b,d,e^			90.5^e^			67.2^b,d,e^		
		Internet users only	93^e^			85.9^b,d,e^			85^b,d,e^			96.2^e^			78.2^b,d,e^		
	**Uses the internet with someone’s help**																
		All adults	6.7^c^			12.4^b,c,d^			13^b,c,d^			3.8^c^			20.7^b,c,d^		
		Internet users only	7.2^e^			14.8^b,c,d^			15.4^b,c,d^			4.1^c^			23.5^b,c,d^		
	**Has an internet-capable device^f^**																
		All adults	94.6^e^			88.6^d,e^			88.5^d^			94.1			91.6^e^		
		Internet users only	99			98.3			97.3			97.6			97.5		
	**Has a desktop, laptop, or tablet computer**																
		All adults	93.9^e^			86.5^b,d,e^			86.3^b,d^			93^e^			85.9^b,d^		
		Internet users only	98.4			96.2			95.7			96.4			92.5^d^		
	Has access to a printer that can be used to print information from the internet or email (internet users only)			90.4			84			86.9			88			86.9	
	**Had a patient portal account by June 2020^g^**																
		All adults	95.7^e^			86.1^b,d,e^			90.1^b,c,d^			97.2			92.7		
		Internet users only	98.4			94.6^b,d^			96.9			98.8			95.9		
	**Sent ≥1 message through the patient portal between January 2019 and June 2020^g^**																
		All adults	67.9^f^			44.1^b,d,e^			54.6^b,d^			68.3			49.1^b,d^		
		Internet users only	72^f^			52.8^b,d,e^			63.2^c^			71.3			52.3^b,d^		
	**Had ≥1 video visit during 2020 (whether the user had ≥1 outpatient visit in 2020)^g^**																
		All adults	50.7^f^			46			51.3^f^			56.7			51.5		
		Internet users only	53^f^			51.3			56^f^			57.3			53.6		

^a^Estimates are based on 2020 Member Health Survey data weighted to the age, sex, and racial and ethnic composition of the health plan’s adult membership in 2019. Unweighted Ns for racial-ethnic groups are as follows: n=2867 White adults, n=225 Chinese adults, n=306 Black adults, n=343 Latino adults, and n=242 Filipino adults and internet users: n=2655 White adults, n=213 Chinese adults, n=255 Black adults, n=286 Latino adults, and n=206 Filipino adults. Survey respondents are a subset of the adults in the electronic health record study cohort.

^b^Prevalence significantly (*P*<.05) differs from that of Chinese adults after adjusting for age and sex.

^c^Within racial and ethnic groups, the percentage is significantly (*P*<.05) higher among those aged 76 to 85 years versus those aged 65 to 75 years after adjusting for sex (data not shown).

^d^Percentage significantly (*P*<.05) differs from that of White adults after adjusting for age and sex.

^e^Within racial and ethnic groups, the percentage is significantly (*P*<.05) lower among those aged 76 to 85 years versus those aged 65 to 75 years after adjusting for sex (data not shown).

^f^Computer, tablet, or smartphone.

^g^Patient portal use data for survey respondents were abstracted from their electronic health records.

#### Factors Associated With Digital Information Technology Use

Black, Latino, and Filipino adults aged 65 to 85 years were less likely than both White and Chinese adults to use the internet (with or without help; Table 4). Among internet users, Black, Latino, and Filipino adults were significantly less likely to use the internet without help than both White and Chinese adults.

Black and Latino adults were also less likely to have an internet-capable device than White adults, but this difference was not seen among internet users. Furthermore, Black, Latino, and Filipino adults were less likely than both White and Chinese adults to have access to an internet-enabled computer, and Filipino internet users were less likely than White internet users to have computer access. Among adults who used the internet, 10% to 16% did not have a printer they could use to print information from the internet or emails. Among all 5 racial and ethnic groups, adults aged 76 to 85 years were less likely to be internet users and to be able to use the internet without help than those aged 65 to 75 years. Among White, Black, and Chinese adults, those aged 76 to 85 years were also less likely to have an internet-capable device than those aged 65 to 75 years.

#### Demographic and Internet Use Factors Associated With Differences in Patient Portal Use

Similar to the full EHR cohort, Black and Latino adults in the survey population were less likely to have a patient portal account than both White and Chinese adults. However, in contrast to the EHR cohort, Filipino adults did not significantly differ from either White or Chinese adults in the survey cohort (Table 4 and model 1 in Table 5). Moreover, similar to the EHR cohort, Black, Latino, and Filipino adults were less likely to have sent a message to their physician through the patient portal than both White and Chinese adults (from January 2019 to June 2020).

**Table 5:** Prevalence ratios showing racial and ethnic group differences in percentages of adults aged 65 to 85 years in the survey population who sent ≥1 secure message, after adjusting for demographic and internet use characteristics.^a^

Model			Reference group		
			White adults, aPR^b^ (95% CI)		Chinese adults, aPR (95% CI)
**Model 1: adjusted for age^c^ and sex**					
	White adults	Reference		1.02 (0.92-1.13)	
	Black adults	0.66 (0.58-0.75)		0.67 (0.58-0.79)	
	Latino adults	0.81 (0.73-0.90)		0.83 (0.72-0.95)	
	Chinese adults	0.98 (0.89-1.09)		Reference	
	Filipino adults	0.72 (0.62-0.83)		0.73 (0.62-0.86)	
**Model 2: adjusted for age^c^, sex, and education^d^**					
	White adults	Reference		1.05 (0.94-1.16)	
	Black adults	0.69 (0.60-0.78)		0.72 (0.61-0.84)	
	Latino adults	0.87 (0.78-0.97)		0.91 (0.79-1.05)	
	Chinese adults	0.95 (0.86-1.06)		Reference	
	Filipino adults	0.71 (0.62-0.82)		0.75 (0.63-0.89)	
**Model 3: adjusted for age^c^, sex, education^d^, and internet use**					
	White adults	Reference		1.04 (0.94-1.14)	
	Black adults	0.73 (0.64-0.82)		0.75 (0.65-0.88)	
	Latino adults	0.89 (0.81-0.99)		0.93 (0.81-1.06)	
	Chinese adults	0.97 (0.88-1.07)		Reference group	
	Filipino adults	0.75 (0.65-0.86)		0.78 (0.65-0.92)	

^a^For these analyses, demographic and internet use data were collected from the 2020 Kaiser Permanente Northern California Member Health Survey and were linked with electronic health record data regarding secure message use from January 2019 through June 2020. Respondent data have been weighted to reflect the age, sex, and racial and ethnic composition of the adult health plan membership in 2019. The aPR estimates the percentage of adults in a racial or ethnic group who sent ≥1 secure message during the study period compared to the percentages of White or Chinese adults who did so, after adjusting for covariates using log-Poisson regression models.

^b^aPR: adjusted prevalence ratio.

^c^Age in 5-year intervals (65-69, 70-74, 75-79, and 80-85 years).

^d^Education is a 3-level variable, with ≤ a high school graduate and some college or associate degree compared to ≥ a bachelor’s degree.

Among all adults, after adjusting for age, sex, education, and racial and ethnic groups, internet users were nearly 70% more likely to have a patient portal account than nonusers (aPR 1.66, 95% CI 1.51-1.83) and >4 times more likely to have sent a message through the patient portal (aPR 4.18; 95% CI 3.19-5.48) than nonusers. Within individual racial and ethnic groups, after adjusting for age, sex, and education, internet users were 40% to 60% more likely to have a patient portal account than nonusers (White users: aPR 1.62, 95% CI 1.45-1.82; Black users: aPR 2.09, 95% CI 1.47-2.97; Latino users: aPR 1.78, 95% CI 1.35-2.35; Filipino users: aPR 1.41, 95% CI 1.11-1.79; and Chinese users: aPR 1.42, 95% CI 0.91-2.21; *P*=.12) and >3 times more likely to have sent a message through the patient portal (White users: aPR 4.33, 95% CI 3.12-6.02; Black users: aPR 5.70, 95% CI 2.35-13.84; Latino users: aPR 5.12, 95% CI 2.11-12.40; Chinese users: aPR 3.78, 95% CI 1.06-13.46; and Filipino users: aPR 1.76, 95% CI 0.87-3.55; *P*=.11). Educational attainment was not associated with having a portal account or with sending a message through the portal after controlling for internet user status.

As presented in Table 5 (models 2 and 3), adjusting for internet user status, in addition to age, sex, and education, did not reduce racial and ethnic group differences in the use of the patient portal to send a message to a health care provider. Black, Latino, and Filipino adults were less likely to have sent a portal message than White adults, and Black and Filipino adults were less likely to have sent a portal message than Chinese adults.

#### Demographic and Internet Factors Associated With Video Visit Use in 2020

Approximately half (46% to 57%) of the older adults in the survey population who had at least 1 outpatient visit from a department that offered video visits had ≥1 video visit during 2020, and similar to the full EHR cohort, no significant racial and ethnic group differences were observed. After adjusting for age, sex, and education, being an internet user was associated with approximately 2-fold greater use of video visits in the overall study population (aPR 2.28, 95% CI 1.79-2.90), but analyses by racial and ethnic groups found that this difference was only among White (aPR 2.71, 95% CI 1.91-3.85), Black (aPR 3.21, 95% CI 1.45-7.11), and Latino (aPR 1.85, 95% CI 1.09-3.15) adults. After adjusting for age, sex, and internet use, educational attainment was neither significantly associated with video visit use in the overall study population nor any of the racial or ethnic groups.

## Discussion

### Principal Findings

In this study, we examined how the use of a health plan patient portal and video visit use differed across racial and ethnic groups in a population of insured older adults whose preferred spoken language was English and who had access to the same health plan patient portal during 2019 and 2020. Using EHR-derived data, we documented lower use of patient portal functions (sending messages and viewing laboratory test results) among Black, Latino, Filipino, Korean, and Vietnamese older adults compared to similarly aged White and Chinese adults; similar use of these patient portal functions among Chinese, Japanese, and South Asian older adults compared to White adults; and lower use of these patient portal functions among older adults (those aged 76 to 85 years) compared to younger (those aged 65 to 75 years) groups. We then used survey information for a subset of White, Black, Latino, Chinese, and Filipino respondents in the EHR cohort to explore potential factors that might contribute to lower engagement in patient portal activities observed in EHR cohort analyses for Black, Latino, and Filipino adults compared to White and Chinese adults.

Overall and for both age groups, Black, Latino, and Filipino adults were less likely to have an activated patient portal account and less likely to have sent messages and viewed laboratory results using their portal account than White adults. Korean, Vietnamese, and South Asian adults did not differ from White adults in having a portal account but were less likely to have used the portal for sending messages and viewing laboratory results. Chinese and Japanese adults did not generally differ from White adults on any of the portal use outcomes, but older Japanese adults (aged 76 to 85 years) were less likely to send secure messages than White adults.

Furthermore, our analyses of racial and ethnic differences in secure messaging and viewing of laboratory results by patients with an activated patient portal account showed that these differences were not simply associated with the lack of an activated patient portal account, although age group differences were attenuated in some cases. Racial and ethnic variations in the video visit use were mainly evident in the 76 to 85 age group, where Black and Latino adults were less likely and Chinese and South Asian adults were more likely to have had a video visit than White adults.

The survey data elucidated factors that might account for the lower patient portal use among Black, Latino, and Filipino adults compared to White and Chinese adults. Black and Latino adults had lower educational attainment than White, Filipino, and Chinese adults, which has been identified as a barrier to the use of the internet and patient portals among older adults in previous research [31,35,46]. Black, Latino, and Filipino adults were less likely to be internet users and to have an internet-enabled computer or tablet, and Black and Latino adults were less likely than White and Chinese adults to have any internet-capable device. While Black, Latino, and Filipino adults who were internet users were more likely than nonusers to have a patient portal account and to have sent a message using the portal, their use of patient portal messaging remained lower than that among White and Chinese adults after controlling for internet user status. Finally, we found that not being an internet user was associated with a lower prevalence of video visit use for older Black and Latino adults, but not for White, Chinese, and Filipino adults.

Our study results are consistent with previous research that found lower rates of patient portal use among US Black, Latino, and Asian older adults compared to White adults [26-30] and among older versus younger Medicare-age adults. Importantly, our study provides novel and contemporary information about racial and ethnic and age group differences in patient portal and video visit use among US Medicare-age adults whose primary language is English. A major contribution is the provision of disaggregated data for Asian ethnic groups, demonstrating variation in the patient portal and video visit use among Asian ethnic groups and identification of factors that may contribute to a lower portal use among the selected ethnic groups.

The survey data for a subset of adults in the EHR cohort suggest that the lower percentages of Black, Latino, and Filipino older adults using patient portal functions compared to White and Chinese adults may in part be due to their lower overall internet use. Internet user status is a barrier to patient portal use that could potentially be removed by providing free or subsidized laptop or tablet computers and printers along with Wi-Fi or data plans for those who do not use the patient portal due to financial barriers. In addition, training on how to use the internet and patient portal for those who lack these skills can be offered. However, the survey data also suggest that factors beyond access to and skills in using the internet may contribute to the observed demographic disparities in patient portal use, especially among adults aged >75 years who do not have as much experience as adults aged 65 to 75 years in using digital information technologies. These factors could include how comfortable and articulate the patient is with composing written digital communications, how important it is to the patient to be able to ask follow-up questions during clinical interactions, and the extent to which patients feel that solely exchanging written messages decreases their sense of interpersonal connection with health care providers [35,47,48].

The disparities we identified in patient portal use and internet use capabilities have implications for a US health care system that is increasingly shifting to using patient portals to share health-related information with patients and to enable bidirectional communication between patients and their health care team. Specifically, to increase patient portal use by Medicare-age adults and to reduce racial, ethnic, and age-related disparities, health plans and community-based institutions, such as libraries and older person centers, should offer skill training, technical support and written instructions, and ongoing encouragement specific to performing patient portal activities for adults who do not use the patient portal on their own but want to learn [20,31,49,50]. This recommendation is supported by a 2023 national survey that found that 24% of older adults whose health plan had a patient portal thought it would be helpful to receive training or a tutorial on patient portal use [48]. Health plans may need to consider loaning or subsidizing the purchase of a low-end internet-enabled computer or tablet with accessories for older adults who do not own one. Research has shown that many older adults find it easier to complete online tasks that involve entering information using a computer with a keyboard and mouse, rather than a touch screen tablet or smartphone [51].

In the first full year of the COVID-19 pandemic, only half of the adults aged 65 to 85 years who had an outpatient visit opted to have a video visit, with lower percentages in the 76 to 85 years age group and among Black and Latino adults compared to White adults in that older age group. While we cannot determine whether the lack of video visits is due to patient preference or digital health access barriers, Medicare and other health insurers should consider parity for phone and video visit reimbursement so that health care systems and clinicians are not financially incentivized to schedule video or in-clinic visits rather than phone visits if doing so is not aligned with patient preferences.

Finally, we want to clearly state that lower use of patient portal functions and video visits by some racial and ethnic groups and by adults aged >75 years should not be construed as lower access to care. Patients are able to get messages to health care providers by contacting the health plan’s regional appointment and advice call center and are able to obtain laboratory test results by a system-generated mailed letter or by requesting a call from their health care team. In addition, patients who had an outpatient visit but not a video visit would have had that visit by phone or in person.

### Limitations and Strengths

Our study has several limitations. First, our EHR cohort was restricted to KPNC members whose preferred language for written materials was English. This restriction was by design because an adult unable to read English and without a proxy user who can read English would have had difficulty interacting with the existing patient portal, which had only basic Spanish text as a second web page language, no functionality in other non-English languages, and limited ability to send secure messages in languages other than English. However, the exclusion of those whose preferred written language is not English limits the generalizability of the study results to adults who can read and understand written English well. Previous studies found large disparities in the use of patient portals and video visits between adults with limited English proficiency and adults with English as their preferred language [52-57]. Our analyses also did not account for health status or recent interactions with the health care system, which have both been shown to influence patient portal access and use [30]. Another limitation is that we had survey data for relatively small samples of Black, Latino, Chinese, and Filipino adults, limiting the statistical power to examine differences between racial and ethnic groups, and no survey data for other Asian ethnic groups. Furthermore, we lacked survey data to examine factors beyond educational attainment and access to digital devices and the internet that have been shown to influence and contribute to patient portal use, including competency and comfort in accessing information on the internet, eHealth literacy, privacy and security concerns, patient communication preferences, physical and cognitive limitations, and availability of high-speed broadband internet in the home [3]. Finally, the study population was restricted to members of 1 Northern California health plan with an older adult membership that is better educated than the general older adult population and that serves a geographic area of the United States that has relatively good high-speed broadband access [35].

However, our study also has several strengths, including a sociodemographically diverse EHR cohort with very large numbers of adults in each of the 9 racial and ethnic subgroups examined. These large subgroup sizes enabled us to document the prevalence of patient portal engagement and video visit use across racial and ethnic groups and by age group within racial and ethnic groups. We believe that this is the first study to document and compare patient portal and video visit use data for such large groups of older adults among 6 different Asian ethnicities and to be able to additionally compare findings across 2 age strata within each ethnic group. In doing so, we found that reporting engagement with patient portal functions and video visit use in just the aggregate 65 to 85 years age group in many instances masked substantially lower engagement with these portal functions in the 76- to 85-year versus 65- to 75-year age group (for all racial and ethnic groups except South Asian group). Another study strength is the examination of patient portal and video visit use based on EHR data rather than self-report and the examination of demographic differences for 3 patient portal outcomes. Finally, we used contemporaneous survey data from the same study population to explore associations of educational attainment and internet access factors with sending messages using the patient portal and having video visits.

### Conclusions

Racial and ethnic group and age group differences in patient portal and video visit use persisted in 2020 in a community-dwelling Medicare-age population that had access to the same patient portal and received care from the same health care delivery system. Contemporary survey data suggested that internet use factors may contribute to lower use among older Black, Latino, and Filipino adults compared to White and Chinese adults. Patient portal use also varied across Asian ethnic groups, underscoring the importance of disaggregating data by Asian ethnicity. Our research highlights the importance of health care providers assessing older adults’ capabilities for engaging with patient portals and video visits, providing training and ongoing technical support to those who are not currently using the patient portal or those who want to improve their navigation skills, making digital equipment accessible when needed, and maintaining nondigital health modalities for health-related communications, including letters, hard copy information (eg, visit summaries, results, instructions, and benefit information), and phone visits. Future research is needed to identify cost-effective ways to reduce racial and ethnic disparities and age-related disparities in the use of patient portals and video visits among older adults and to serve older adults with limited English proficiency.

## Appendix

Multimedia Appendix 1 Forest plots showing prevalence ratios comparing patient portal outcomes (non-White racial and ethnic groups to White adults and non-Chinese Asian ethnic groups to Chinese adults) after adjusting for age and sex.

## Abbreviations

aPR: adjusted prevalence ratio
EHR: electronic health record
KPNC: Kaiser Permanente Northern California
