# Supplementary material for: Examining Whether Patient Portal and Video Visit Use Differs by Race and Ethnicity Among Older Adults in a US Integrated Health Care Delivery System: Cross-Sectional Electronic Health Record and Survey-Based Study
Source: JMIR Aging. 2024 Nov 7;7:e63814. doi: 10.2196/63814 (PMC11582487; doi:10.2196/63814)
Supplement: Multimedia Appendix 1 [file aging_v7i1e63814_app1.pdf]

Multimedia Appendix

Figure 1: Prevalence ratios for activated patient portal account by end of December 2020, comparing (a) eight racial and ethnic groups to White adults and (b) five Asian ethnic groups to Chinese adults

Figure 2: Prevalence ratios for having sent ≥ 1 secure message during 2019–2020, comparing (a) eight racial and ethnic groups to White adults and (b) five Asian ethnic groups to Chinese adults

Figure 3: Prevalence ratios for having viewed ≥ 1 lab test result during 2020, comparing (a) eight racial and ethnic groups to White adults and (b) five Asian ethnic groups to Chinese adults

Figure 4: Prevalence ratios for having ≥ 1 video visit during 2020, comparing (a) eight racial and ethnic groups to White adults and (b) five Asian ethnic groups to Chinese adults

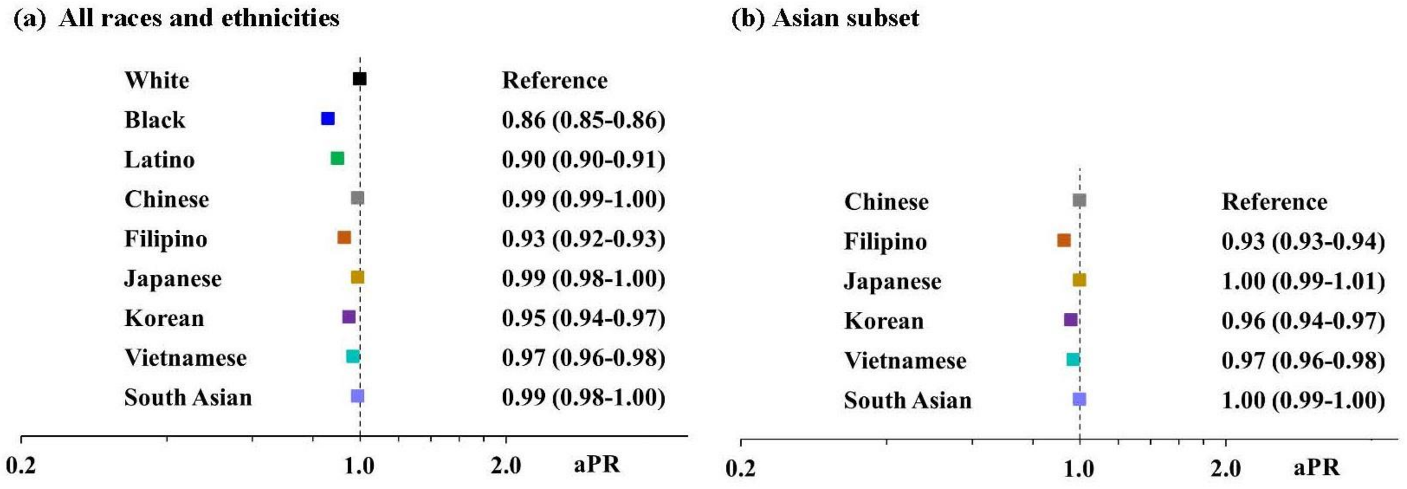

Each forest plot represents a modified log-Poisson model that adjusts for age and sex. Non-Hispanic White (White) was used as the reference group for model (a) and Chinese was used as the reference group for model (b). Adjusted prevalence ratios are reported with 95% confidence intervals.

Figure 1: Prevalence ratios for activated patient portal account by end of December 2020, (a) comparing eight racial and ethnic groups to White adults and (b) comparing five Asian ethnic groups to Chinese adults.

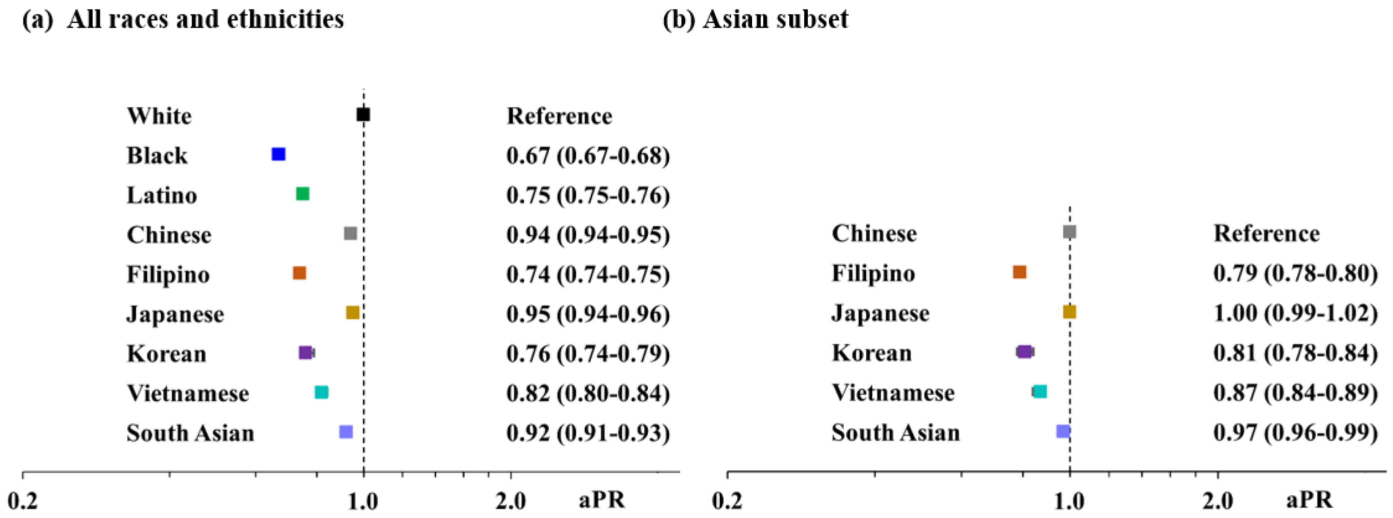

Each forest plot represents a modified log-Poisson model that also includes age and sex. Non-Hispanic White (White) was used as the reference group for model (a) and Chinese was used as the reference group for model (b). Adjusted prevalence ratios are reported with 95% confidence intervals.

Figure 2: Prevalence ratios for having sent ≥1 secure message during 2019-2020, (a) comparing eight racial and ethnic groups to White adults and (b) comparing five Asian ethnic groups to Chinese adults.

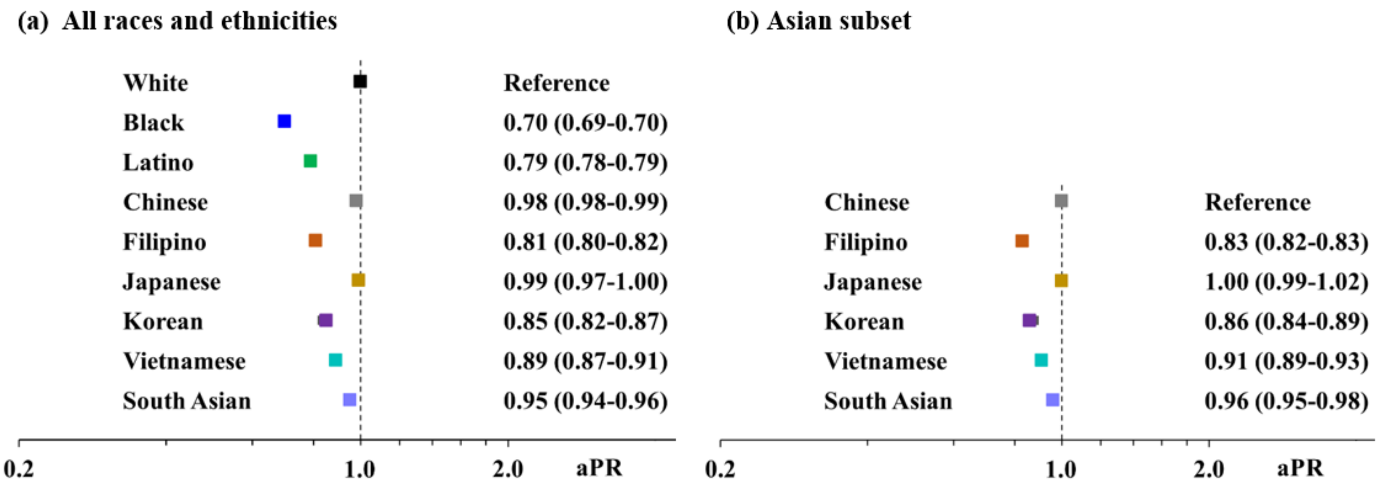

Each forest plot represents a modified log-Poisson model that also includes age and sex. Non-Hispanic White (White) was used as the reference group for model (a) and Chinese was used as the reference group for model (b). Adjusted prevalence ratios are reported with 95% confidence intervals. Analyses were restricted to adults who had ≥1 lab test result released during 2020.

Figure 3: Prevalence ratios for having viewed ≥1 lab test result during 2020, (a) comparing eight racial and ethnic groups to White adults and (b) comparing five Asian ethnic groups to Chinese adults.

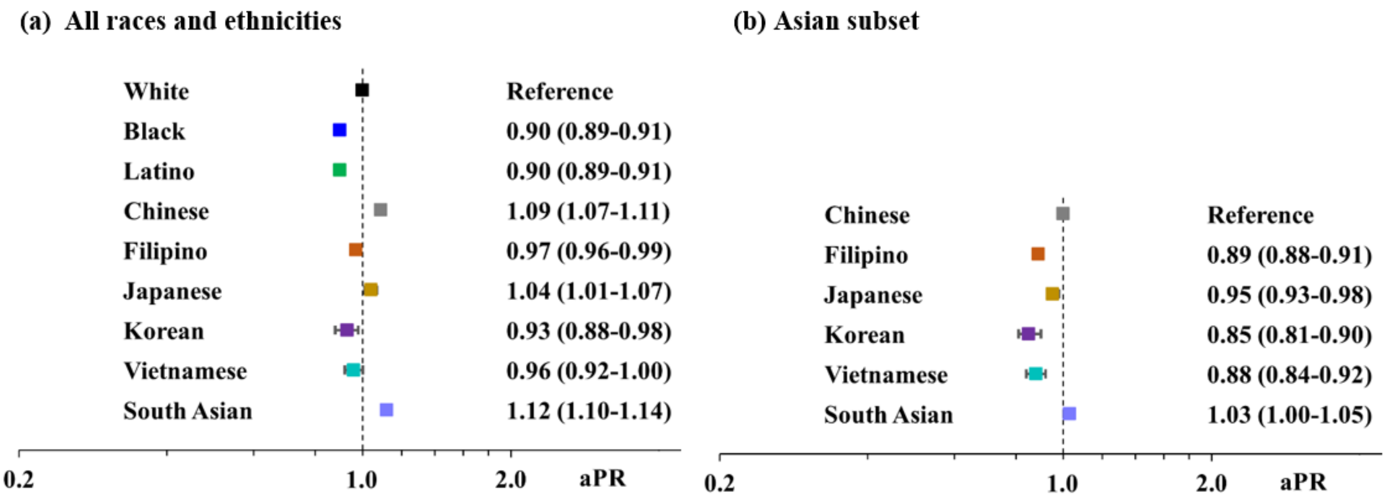

Each forest plot represents a modified log-Poisson model that also includes age and sex. Non-Hispanic White (White) was used as the reference group for model (a) and Chinese was used as the reference group for model (b). Adjusted prevalence ratios are reported with 95% confidence intervals. Analyses were restricted to adults who had ≥1 outpatient visit in 2020, in a department that offered video visits.

Figure 4: Prevalence ratios for having ≥1 video visit during 2020, (a) comparing eight racial and ethnic groups to White adults and (b) comparing five Asian ethnic groups to Chinese adults.
